# Understanding Multilevel Correlates of Long-Term Physical Activity Trajectories Among Middle-Aged and Older Adults: A Machine-Learning Analysis

**DOI:** 10.3390/bs16071210

**Published:** 2026-07-17

**Authors:** Wenjing Liu, Jiao Liu, Yunru Shao, Lu Guo

**Affiliations:** 1 School of Psychology, Beijing Sport University, Beijing 100084, China; psy2023ash@bsu.edu.cn (W.L.); shaoyr@bsu.edu.cn (Y.S.); 2 School of Sport and Art Education, Beijing Institute of Education, Beijing 100120, China; liujiao@bjie.ac.cn

**Keywords:** physical activity, machine learning, trajectory analysis, social ecological model, middle-aged and older adults

## Abstract

Background: Physical activity (PA) in later life is intertwined with complex, multilevel factors and represents an important behavioral science topic. This study examined heterogeneous long-term PA patterns among middle-aged and older adults and explored how multilevel baseline variables organized by the Social Ecological Model (SEM) contributed to trajectory classification. Methods: Longitudinal data were drawn from the China Health and Retirement Longitudinal Study (CHARLS). Repeated PA measures were modeled using latent class growth modeling and growth mixture modeling to identify distinct trajectory groups. The resulting trajectory membership was then used in exploratory machine-learning classification analyses, in which SEM-organized baseline variables were evaluated according to their relative contribution to classification. Results: Three PA trajectories were identified: persistently low PA, high-decreasing PA, and moderate-increasing PA. Among the evaluated classifiers, the random forest showed the highest internal classification performance, with AUC values ranging from 0.943 to 0.945. Social participation, age, educational level, and gender showed the most consistently high variable-importance rankings across the two random-forest classification tasks. Conclusions: The findings highlight that long-term PA patterns among middle-aged and older adults do not follow a single uniform trajectory but instead show substantial trajectory heterogeneity. Multilevel factors organized within the Social Ecological Model showed exploratory value for classifying distinct PA trajectories, suggesting that future PA research should move beyond overall activity levels and further consider individuals’ dynamic trajectory contexts and their corresponding classification-relevant features.

## 1. Introduction

As population aging accelerates worldwide, promoting healthy aging has become an urgent public health priority. According to the World Health Organization, by 2050, the number of individuals aged 60 years and older is projected to reach 2.2 billion, constituting nearly a quarter of the total population. In China, this proportion is expected to exceed one-third, further underscoring the urgency of promoting healthy aging (Tong, 2021). Among various strategies to promote healthy aging, PA is widely acknowledged as a modifiable behavior that helps maintain functional ability, prevent chronic diseases, enhance quality of life, and improve psychological well-being (Zlatar et al., 2019). Regular PA has also been associated with fewer depressive symptoms, higher subjective well-being, and reduced aging-related health risks among middle-aged and older adults (W. Chen et al., 2022).

Although previous studies have examined correlates of PA levels among middle-aged and older adults, the long-term patterns of PA change remain insufficiently understood. PA is not a static behavior: although individuals who are active in midlife are more likely to remain active in later life, overall PA tends to decline with advancing age, and this decline does not occur uniformly across individuals (Aggio et al., 2018; Fang et al., 2023). A systematic review of 27 longitudinal studies found that three- or four-class trajectory solutions were most common. Among late-middle-aged and older adults, inactive or low-active trajectories frequently comprised the largest subgroups, and individuals following declining trajectories often approached the activity levels of persistently inactive groups over time (Lounassalo et al., 2019). Evidence from China further demonstrates this heterogeneity. Using longitudinal CHARLS data, Fang et al. (2023) identified three distinct patterns among older Chinese adults: sustained low PA, moderate PA with gradual decline, and sustained high PA with substantial fluctuations, with the gradual-decline trajectory accounting for the majority of participants. Longitudinal research conducted during major later-life transitions also suggests that PA change may be stage-specific rather than linear, with short-term increases in walking or other moderate-intensity activities sometimes followed by subsequent declines during later follow-up (Van Dyck et al., 2016; Stenholm et al., 2016). Collectively, these findings indicate that PA among middle-aged and older adults is characterized by an overall age-related decline, substantial between-person heterogeneity, and stage-dependent fluctuations. This complexity underscores the need to identify distinct long-term PA trajectories and the factors that distinguish membership in these trajectories.

To examine variables associated with distinct PA trajectories, it is necessary to consider the multilevel factors that may be associated with PA participation and maintenance. The Social Ecological Model (SEM)provides an integrative framework for understanding health behavior by emphasizing that individual behavior is shaped by influences operating at the individual, interpersonal, organizational, community, and policy levels. It also provides a theoretical basis for identifying potential candidate domains for future intervention research across ecological levels (McLeroy et al., 1988; Kerr et al., 2018; Wu & Yu, 2019; Zhang et al., 2020). The SEM has also been used to identify and organize factors associated with PA among older adults (Baert et al., 2016; Boulton et al., 2018). Previous PA trajectory studies have primarily explained differences in trajectory membership in terms of age, sex, education, income, health status, and lifestyle factors (Wang et al., 2024). Similarly, a systematic review found that existing studies most commonly examined sociodemographic characteristics, socioeconomic status, health behaviors, health-related factors, and family or social support, while social capital, environmental factors, and other contextual influences warrant further investigation (Lounassalo et al., 2019). Although the broader PA literature has demonstrated that environmental accessibility, community resources, and rural–urban contexts may influence PA participation, environmental determinants and their joint contributions alongside individual-level factors have received comparatively limited attention (Deng & Paul, 2018; Hobbs et al., 2020). Thus, although previous studies have considered factors from multiple domains, these factors have not been consistently integrated and compared within a unified social ecological framework, limiting understanding of how variables across ecological levels may help distinguish long-term PA trajectory membership (D’Amore et al., 2021). The present study used the SEM to guide the selection and classification relevant variables. The study examines membership in long-term PA trajectories and applies machine learning to compare the relative classification contributions of factors across ecological levels.

Person-centered trajectory approaches have been used to identify heterogeneous patterns of PA change. In the PA literature, group-based trajectory modeling (GBTM; Wang et al., 2024) and latent class growth modeling (LCGM; Fang et al., 2023) have been applied to classify individuals into distinct PA trajectory groups. These approaches are valuable for describing between-class heterogeneity, but they typically assume that individuals within the same class follow a similar developmental pattern and therefore provide limited information about within-class variability. This assumption may be particularly restrictive for PA among middle-aged and older adults because individuals following the same broad trajectory may still differ in their initial PA levels and rates of change. Growth mixture modeling (GMM) relaxes this assumption by allowing individual-level variation in growth parameters within latent classes, thereby capturing both between-class heterogeneity and within-class differences. Previous longitudinal research has used LCGM to identify a plausible latent class structure before fitting GMM to account for within-class variability (H. Chen et al., 2024). Consistent with this sequential approach, the present study first applied LCGM to identify a parsimonious and substantively interpretable class structure and then fitted GMM to the retained solution to account for individual variability within each trajectory class. The resulting GMM-based trajectory membership was used as the outcome in the machine learning analyses.

In recent years, machine learning has been increasingly applied in PA research. Existing studies have primarily used accelerometers, activity-tracker, or continuous physiological data to classify activity types and intensity levels (Willetts et al., 2018; Ahmadi et al., 2020; Thornton et al., 2023), identify temporal patterns of daily activity (Nawrin et al., 2024), and predict daily goal attainment or short-term exercise-related outcomes (Dijkhuis et al., 2018; Tyler et al., 2022). These studies demonstrate the potential of machine learning to analyze complex behavioral and physiological data. However, relatively few studies have treated PA trajectory membership identified from multiyear longitudinal data as a classification outcome, particularly while simultaneously incorporating classification relevant variables across multiple levels of the Social Ecological Model (SEM). Machine-learning methods are well suited to analyzing complex data, detecting latent patterns, and modeling potentially nonlinear relationships without requiring all functional forms to be specified a priori (Rubinger et al., 2023). These capabilities are consistent with the multilevel perspective of the SEM, because factors operating across ecological levels may jointly distinguish PA trajectory membership through nonlinear, conditional, and interactive patterns. Accordingly, the present study employed machine learning as a complementary classification and exploratory approach to evaluate the joint contribution of baseline factors across ecological levels to the classification of long-term PA trajectories.

Using longitudinal data from the CHARLS, the present study pursued three sequential and complementary aims. First, the descriptive aim was to identify and characterize heterogeneous longitudinal PA trajectories among middle-aged and older adults using latent class growth modeling and growth mixture modeling. Second, the classification aim was to develop and internally evaluate five machine-learning models for distinguishing membership in the identified trajectories across two prespecified pairwise classification tasks. Third, the exploratory aim was to use the Social Ecological Model to guide the selection and organization of baseline variables across individual, interpersonal, organizational, and policy-related levels and to evaluate their relative contributions to classification. This exploratory analysis also compared variables that remained salient across both classification tasks with those whose relative importance differed between trajectory contrasts. In general, we seek to contribute to the advancement of knowledge on PA among middle-aged and older adults and provide preliminary insights that may inform future intervention research to promote healthy aging and PA.

## 2. Methods

### 2.1. Participants

The data utilized in this study were derived from the China Health and Retirement Longitudinal Study (CHARLS), a nationwide panel survey that follows Chinese adults aged 45 years and above, along with their spouses, and repeatedly collects information on health status, economic conditions, and social behaviors (Zhao et al., 2012). This study used data from three waves of CHARLS: Wave 1 (2011), Wave 2 (2013), and Wave 4 (2018). The CHARLS study was approved by the Institutional Review Board of Peking University (IRB00001052-11015). The initial CHARLS 2011 sample included 17,705 participants. Because the primary objective of this study was to identify longitudinal PA trajectories, participants were first required to have valid/calculable PA data across all three waves used in the present study, namely 2011, 2013, and 2018. This criterion retained 6887 participants. Participants younger than 45 years were then excluded, leaving 6732 participants. Finally, participants with incomplete or invalid baseline predictor information were excluded to ensure the completeness of the machine-learning analyses, resulting in a final analytical sample of 3112 participants. To further evaluate the potential influence of sample exclusion, we compared available baseline characteristics between participants included in and excluded from the final analytical sample. The comparison showed that standardized mean differences were generally small. The detailed baseline comparison is presented in Supplementary Table S1.

### 2.2. Outcome Variables

PA was assessed at three waves (2011, 2013, and 2018) and operationalized as total metabolic equivalent (MET) scores to reflect overall PA levels. At each wave, participants were asked to report the frequency and duration of different intensities of PA performed in the past week, including vigorous activities (e.g., carrying heavy loads, farming, fast cycling), moderate activities (e.g., carrying light loads, regular cycling, and household chores), and walking (including work-related, transportation, and recreational walking). Each activity session was required to last at least 10 min. The duration of daily PA was categorized into four levels and coded as an ordinal duration index: 1 for <0.5 h, 2 for 0.5–2 h, 3 for 2–4 h, and 4 for >4 h. For each activity intensity, the weekly duration index score was calculated by multiplying the number of active days per week by the corresponding daily duration category code. The total PA score was then calculated as follows: 8.0 × vigorous activity weekly duration index score + 4.0 × moderate activity weekly duration index score + 3.3 × walking weekly duration index score, following Deng and Paul (2018).

### 2.3. Variables

Baseline variables were derived from the baseline survey (Wave 1, 2011) of CHARLS and were selected to classify PA trajectory membership among middle-aged and older adults. All baseline predictors were coded according to the official CHARLS questionnaire and coding manual. The selection of variables was guided by the SEM and existing literature on PA, ensuring the inclusion of multilevel factors associated with PA behaviors. We categorized these factors within the theoretical framework of the social–ecological model (McLeroy et al., 1988; Sallis et al., 2006) and the application and improvement of other researchers (Han et al., 2023; Peng et al., 2021). A total of 12 variables were included and categorized into four levels according to the SEM framework: (1) individual level (age, gender, educational level, self-reported health status, depressive symptoms, sleep, and life satisfaction); (2) interpersonal level (marital status, child support, and social participation); (3) organizational level (retirement status, reflecting employment-related organizational involvement); and (4) societal/policy level (subsidies, reflecting policy-related financial support).

### 2.4. Data Analysis

**Figure 1:**
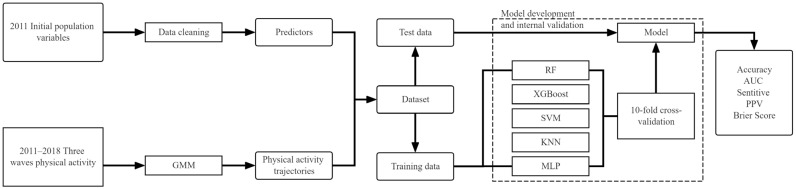
Research process flowchart for the entire study.

The primary statistical analysis encompassed two pivotal phases (see Figure 1). For the trajectory analysis, the 2011, 2013, and 2018 assessment waves were coded as 0, 1, and 2, respectively, to capture the heterogeneity in PA patterns across the three waves. Total PA scores (MET values) at each wave were treated as longitudinal indicators and entered into LCGM and GMM to identify distinct trajectory patterns. Each participant was assigned to a trajectory group based on the maximum posterior probability. Previous research recommends conducting LCGM prior to GMM to determine the optimal number of trajectory classes (H. Chen et al., 2024). LCGM was first applied as an exploratory approach, assuming no within-class variability, followed by GMM to account for individual differences within each trajectory group. The optimal number of trajectory classes was determined based on model fit indices, including the Sample-Size Adjusted Bayesian Information Criterion (SABIC), entropy, Bootstrap Likelihood Ratio Test (BLRT), and Vuong–Lo–Mendell–Rubin Likelihood Ratio Test (VLMR-LRT), as well as the interpretability of the classes. All trajectory models were estimated using Mplus 8.3 (Muthén & Muthén, Los Angeles, CA, USA). The identified trajectory groups were then used as the outcome variable for the second phase of analysis.

In the second phase, ML methods were applied to evaluate the relative contribution of baseline variables to PA trajectory classification. The final ML dataset included 3112 participants and 12 baseline variables. Missing and invalid values were handled during variable construction and sample selection; therefore, no statistical imputation was performed during model development. All variables were numerically coded according to the CHARLS questionnaire and coding manual. The persistently low PA trajectory was selected as the common reference group, and two pairwise binary classification tasks were conducted: high-decreasing PA versus persistently low PA and moderate-increasing PA versus persistently low PA. The sample with assigned trajectory labels was split using stratified 70/30 sampling with fixed random-state settings. To reduce the risk of information leakage, SMOTE was not applied to the full dataset before model evaluation. Data standardization was fitted only on the training subset using StandardScaler, and SMOTE was subsequently applied only to the standardized training subset to address class imbalance, with these steps performed prior to the repeated 10-fold stratified cross-validation procedure. Five ML models were compared: RF, XGBoost, SVM, KNN, and MLP. Model performance was assessed using 10-fold stratified cross-validation with repeated runs and evaluated using accuracy, AUC, sensitivity, PPV, and Brier score. No data-driven hyperparameter tuning was conducted; models were implemented using predefined/default settings, with fixed random-state settings used where applicable. All analyses were conducted using Python 3.11.7 with the Scikit-learn version 1.6.1, imbalanced-learn version 0.13.0, and XGBoost packages version 2.1.1.

## 3. Results

LCGM revealed the trajectory of PA, with clustering numbers ranging from 2 to 6 (Table 1). The results showed that as the number of trajectory patterns increased, the sample-adjusted Bayesian Information Criterion (SABIC) decreased. Although the four-class LCGM solution showed improvement in some statistical fit indices, the additional class mainly represented a further subdivision of an existing trajectory according to its level and magnitude of decline and followed a similar overall direction of change, rather than identifying a qualitatively distinct longitudinal pattern. Based on model fitting indicators, classification clarity, and theoretical interpretability, the three-class Growth Mixture Model (GMM3) was selected as the optimal model (SABIC = 112,226.326, entropy = 0.853, VLMR-LRT *p* < 0.001, BLRT *p* < 0.001), with the minimum category accounting for 16.1% of the sample. This optimal model demonstrated robust classification quality; specifically, the average posterior probabilities for the “high-decreasing PA,” “moderate-increasing PA,” and “persistently low PA” classes were 0.869, 0.918, and 0.958, respectively. Additionally, the corresponding odds of correct classification were 33.20, 57.68, and 11.21, with class-specific uncertainty values of 0.131, 0.082, and 0.042. These metrics collectively indicate excellent separation among the three trajectory classes, thereby validating the use of the most likely class assignments as outcome labels in subsequent machine-learning analyses.

**Figure 2:**
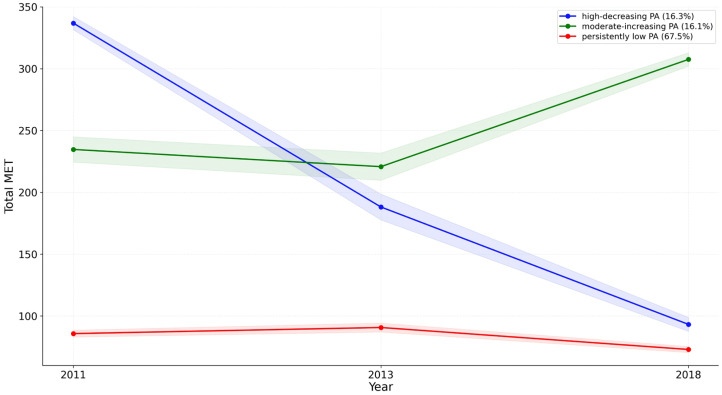
Physical activity trajectories.

According to the characteristics of trajectory changes, PA trajectories are divided into three patterns, namely: (1) persistently low PA (67.5%), which maintains a low level of PA at each measurement point; (2) high-decreasing PA (16.3%), which showed a generally decreasing pattern across the observed waves; (3) moderate-increasing PA (16.1%), which showed a moderate level followed by an increase at the final measurement occasion, with each pattern displaying a unique trajectory of PA (Figure 2).

At the trajectory level, the three identified groups showed distinct patterns of PA across the three measurement occasions. The high-decreasing PA trajectory (16.3%, *n* = 508) had the highest PA level in 2011 (*M* = 336.84, *SE* = 2.70), followed by lower levels in 2013 *(M* = 188.16, *SE* = 5.31) and 2018 (*M* = 93.21, *SE* = 2.87), indicating a generally decreasing pattern across the observed waves. The moderate-increasing PA trajectory (16.1%, *n* = 502) showed a moderate PA level in 2011 (*M* = 234.69, *SE* = 5.22), a similar level in 2013 (*M* = 220.77, *SE* = 5.61), and a higher level in 2018 (*M* = 307.51, *SE* = 2.76), suggesting an overall increasing pattern by the final measurement occasion. In contrast, the persistently low PA trajectory comprised the largest proportion of participants (67.5%, *n* = 2102) and showed consistently low PA levels across the three waves, with mean values of 85.75 (*SE* = 1.41) in 2011, 90.67 (*SE* = 1.84) in 2013, and 72.91 (*SE* = 1.25) in 2018.

As shown in Table 2, significant differences in demographic and social characteristics were observed across the three PA trajectories. Social participation, child support, subsidy receipt, retirement status, depressive symptoms, and life satisfaction also differed significantly across trajectory groups (*p* < 0.05). No significant differences were found in self-reported health status or sleep (*p* > 0.05). Notably, although the persistently low PA group had the highest mean social participation score, this descriptive result should not be considered inconsistent with the relatively high importance of social participation in the machine-learning analysis. Descriptive statistics reflect unadjusted between-group differences in the means, proportions, and distributions of variables, whereas machine-learning feature importance reflects the relative contribution of each predictor to trajectory classification when all variables are considered jointly and does not indicate the direction of the association.

Based on the identified trajectory classifications, the persistently low PA trajectory, which represented the long-term insufficient-PA pattern of primary interest, was selected as the common reference group, and two pairwise binary classification tasks were conducted. Five common ML methods were employed for comparative analysis, including RF, XGBoost, SVM, KNN, and MLP. The specific performance of each model is presented in Table 3.

**Figure 3:**
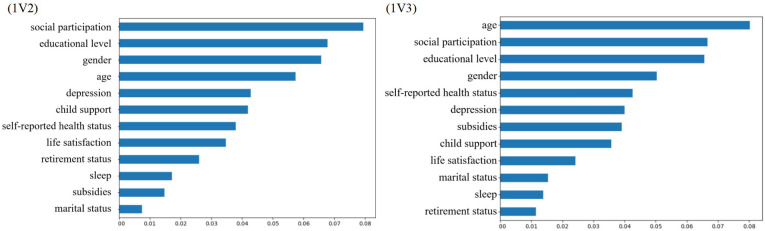
Relative variable-importance rankings for the two PA trajectory classification tasks.

Given that RF showed the highest overall internal classification performance across the two repeated validation tasks, variable-importance results from this model were used descriptively to examine which SEM-organized baseline variables contributed most to trajectory classification. Figure 3 presents the ranked order of variables that contributed most to classification derived from the RF algorithm when applied to the classification tasks of discriminating between distinct PA trajectory patterns.

## 4. Discussion

Using three waves of longitudinal data from CHARLS, this study identified three distinct PA trajectories among middle-aged and older adults. Among the ML models evaluated, RF showed the highest overall internal classification performance in this study. Social participation, educational level, age, and gender showed relatively high classification relevance across the classification tasks.

A systematic review of PA trajectory studies showed that three or four trajectory classes were most commonly identified across the life course and that the proportion of persistently inactive individuals tended to increase with age (Lounassalo et al., 2019). A CHARLS-based study focusing on the retirement transition among older Chinese adults also revealed distinct developmental patterns of PA, including a sustained low level of PA, a middle level of PA with gradual decline, and a sustained high level of PA with significant fluctuations (Fang et al., 2023). These findings suggest that PA behavior does not follow a single homogeneous pattern over time but instead exhibits substantial population heterogeneity. In the present study, the persistent low-level trajectory accounted for the largest proportion of participants, reaching 67.5%, indicating that long-term insufficient PA may be the predominant behavioral pattern in our sample, thereby enriching our understanding of PA dynamics. This finding is in line with the nationally representative cohort study by Wang et al. (2024), which similarly showed that the persistently low PA trajectory accounted for 74.2% of adults aged over 55 years. Although differences in sample age, follow-up waves, PA measurement, and modeling strategies may lead to variations in the number and proportion of trajectory classes across studies, a relatively large low-activity group has been repeatedly identified, highlighting the public health significance of individuals with persistently low PA. To translate the public health relevance of this numerically predominant persistently low PA trajectory into more behavioral relevance, we further examined how multilevel baseline characteristics distinguished individuals in the two alternative trajectory patterns from those who remained persistently low.

Following the identification of the PA trajectories, machine learning models were used to evaluate the joint classification contribution of baseline variables guided by the Social Ecological Model (SEM) to distinguish trajectory membership. RF showed the highest overall internal performance and was therefore used descriptively to assess global variable importance. Conventional regression approaches remain well suited to estimating the direction and magnitude of prespecified associations, whereas the machine-learning analyses in the present study focused on classification performance and the relative contribution of multilevel variables to classification. This analytical distinction is relevant to behavioral research, in which individual, interpersonal, and broader contextual factors may jointly contribute to PA outcomes (Hobbs et al., 2020). Evaluating baseline variables across the two random forest classification tasks revealed that social participation, education level, age, and gender consistently ranked highly, but their relative classification hierarchies varied across the two contrasts. Specifically, social participation was the highest-ranked variable differentiating the high-decreasing PA trajectory from the persistently low PA trajectory, whereas age became more prominent when distinguishing the moderate-increasing PA trajectory from the persistently low PA trajectory. This task-specific pattern is in line with PA-related machine-learning studies showing that behavioral information may emerge from activity patterns and profiles rather than from total activity volume or single variables alone (Nawrin et al., 2024). Using the persistently low PA trajectory as a shared reference group may therefore help identify both common classification-relevant variables and trajectory-specific variation (H. Chen et al., 2024). These findings suggest that dynamic PA trajectories may share certain common baseline characteristics while also showing trajectory-specific multivariable behavioral profiles. Although RF can accommodate nonlinear relationships and interactions (Karanth et al., 2021), this study reports global variable importance rather than thresholds or interaction structures. Therefore, the findings should be interpreted as exploratory classification evidence for hypothesis generation, not as evidence of causal mechanisms or established intervention targets.

At the individual level, age, gender, and educational level showed relatively high classification relevance for PA trajectory membership. These findings are consistent with previous evidence showing that demographic characteristics are closely associated with PA participation among adults and older adults. Population-based evidence underscores that while sex and age are strongly relevant for PA patterns, their expressions are shaped by sociocultural contexts (Kokolakakis et al., 2012). Furthermore, educational level may help distinguish higher- versus lower-dose PA participation contexts, potentially reflecting differences in resources, health literacy, self-efficacy, motivation, and access to PA-related opportunities (R. M. Eime et al., 2018). In the present RF classification tasks, gender ranked highly in the RF classification tasks. This gender-related pattern may align with evidence that structural constraints, such as gendered social roles and unequal resource distribution, can limit older women’s PA opportunities (Zhou et al., 2024). Traditional family caregiving expectations may also reduce women’s discretionary time, which could be relevant to understanding gendered PA patterns in later life (Young & Sharpe, 2016).

Beyond these relatively stable sociodemographic factors, depressive symptoms and self-reported health status represent health-related individual-level variables that may contribute to PA trajectory classification in a multivariable context. Previous studies connect severe depressive symptoms with diminished PA among middle-aged and older adults (Carvalho et al., 2021; Wang et al., 2024). Over time, this erosion precipitates a vicious cycle in which declining PA reciprocally exacerbates depressive symptoms (Kandola et al., 2019). Alongside depressive symptoms, self-reported health status may be relevant to PA trajectory classification because it may capture perceived physical capability and health-related constraints. Aggio et al.’s (2018) 20-year follow-up study similarly reported that declines in PA among older adults occurred alongside changes in health and social functioning. Importantly, this longitudinal decline is often compounded by specific proximal barriers in later life; as noted by Crossman et al. (2024), issues such as chronic disease burden, functional limitations, and a heightened fear of injury frequently intercept and curtail sustained PA maintenance. Ultimately, these findings highlight the complex, multifaceted nature of individual-level PA factors, offering preliminary insights that could help inform future translational practice.

At the interpersonal level, when distinguishing the high-decreasing PA and moderate-increasing PA trajectories from the persistently low PA trajectory, social participation consistently ranked among the top two classification-relevant variables. From a social–ecological perspective, social participation represents a pivotal interpersonal resource, an explanation well-documented across both qualitative syntheses and intervention designs (Franco et al., 2015; Spiteri et al., 2019; Peters et al., 2024). Paradoxically, however, descriptive statistics revealed that the persistent low PA trajectory exhibited the highest overall level of social participation. This seemingly counterintuitive divergence highlights the limitation of relying strictly on single-level descriptive analyses and underscores the complex, heterogeneous nature of social engagement. One plausible explanation is the multidimensionality of leisure activities. As Bu et al. (2024) emphasized, leisure engagement is an umbrella construct spanning socially, cognitively, and physically oriented behaviors. The broad measurement of social participation in CHARLS aggregates physically active formats (e.g., sports or qigong clubs) with sedentary or low-intensity activities (e.g., playing mah-jong, chess, or cards). Crucially, different facets of social involvement yield distinct behavioral outcomes; for instance, while close social ties primarily enhance mood, encounters with peripheral ties are more uniquely coupled with objectively measured PA (Fingerman et al., 2020). Consequently, the high social participation observed in the persistent low PA group likely reflects broader emotional connectedness or involvement in sedentary leisure rather than active physical engagement. This may partly account for why social participation remained highly informative in the multilevel RF model while appearing paradoxically elevated in descriptive group-level profiles—a critical distinction between interindividual classification dynamics and intraindividual fluctuations (Knapova et al., 2024). Therefore, the higher social participation observed in the persistent low PA group should be understood as a group-level association rather than evidence that social participation directly alters PA within individuals. Future research should explicitly differentiate physically active forms of social participation from sedentary, cognitive, and community-based variants to clarify their distinct implications for late-life PA trajectories.

To our knowledge, this study represents an early attempt to use machine-learning methods to examine the classification of PA trajectory membership among middle-aged and older adults. The RF variable-importance rankings may provide trajectory-specific clues for future PA intervention research. First, the persistently low PA trajectory comprised the largest subgroup and represents the central public health challenge of long-term insufficient PA, suggesting that PA initiation and barrier reduction remain the relevant priorities for future research for this group. Second, the high-decreasing PA trajectory was characterized by a high initial PA level followed by a subsequent decline, indicating that this group may not lack a PA foundation but may instead face difficulties in PA maintenance. In the RF binary comparison, social participation was the most prominent variable distinguishing the high-decreasing PA trajectory from the persistently low PA trajectory, suggesting that future research on PA maintenance and decline prevention could further examine the role of social participation. However, the focus should not be on increasing social participation in general, but on distinguishing physically active social participation from sedentary social participation and testing their differential roles in PA maintenance. Finally, the moderate-increasing PA trajectory reflected a positive upward PA pattern. In this RF comparison, age was more prominent in distinguishing the moderate-increasing PA trajectory from the persistently low PA trajectory. Because age is not modifiable, it may serve as an observational marker, whereas social participation and self-reported health status may represent more modifiable candidate domains for future research on the maintenance of positive PA change.

This study has several limitations. First, despite generally small standardized mean differences between included and excluded participants at baseline, selection bias resulting from the exclusion of individuals with missing longitudinal PA or baseline predictor data cannot be ruled out when generalizing the findings to the broader Chinese middle-aged and older population. Second, the CHARLS social participation measure combined physically active, sedentary, cognitive, and community-based activities into a single composite score. Consequently, the present analysis could not determine which specific forms of participation contributed to trajectory classification, limiting the directional and practical interpretation of this variable. More fine-grained measures are needed before social participation can be evaluated as a candidate area for PA promotion research. Third, because the PA trajectories were based on only three measurement waves, the stable estimation of complex non-linear changes was inherently limited. Accordingly, the identified trajectory shapes and their corresponding labels can only be interpreted as descriptive approximations rather than as precise estimates of the true rate or functional form of change. In addition, although repeated stratified cross-validation was used to evaluate internal stability, the machine-learning models were not externally validated in an independent cohort. Therefore, the machine-learning results can only be interpreted as exploratory internal classification evidence. Finally, a notable limitation stems from the self-reported nature of the PA assessment, which relied on broad frequency and duration categories. This approach is inherently prone to recall and reporting biases, thereby restricting the precision of the MET-weighted PA scores. Future studies should incorporate objective measurement tools, such as accelerometers or wearable devices, to improve the accuracy of PA assessment.

## 5. Conclusions

Using longitudinal data from CHARLS, this study identified three distinct PA trajectories among middle-aged and older adults in China: persistently low PA, high-decreasing PA, and moderate-increasing PA. Among the evaluated ML models, the random forest showed the highest internal classification performance, with social participation, age, educational level, and gender showing the most consistently high variable-importance rankings across the two classification tasks. These findings extend current knowledge of heterogeneous PA trajectories and their multilevel classification profiles, suggesting that long-term PA among middle-aged and older adults should not be understood as a single uniform pattern but rather as distinct trajectory patterns with potentially different classification-relevant profiles. Future PA research should therefore move beyond overall activity levels and further examine individuals’ dynamic trajectory contexts, trajectory-specific behavioral profiles, and potentially modifiable domains that may be relevant to PA initiation, maintenance, or positive change over time. Overall, integrating PA trajectory modeling with machine-learning approaches within a social–ecological framework may offer a useful exploratory strategy for generating future research questions on healthy aging and PA promotion.

## Figures and Tables

**Table 1:** Results of latent trajectory analysis (LCGM and GMM).

Model	SABIC	Entropy	VLMR-LRT *p* Value	BLRT *p* Value	Smallest Class
LCGM					
LCGM2	112,761.143	0.894	<0.001	<0.001	0.188
LCGM3	112,414.577	0.853	<0.001	<0.001	0.161
LCGM4	111,939.028	0.881	0.003	<0.001	0.087
LCGM5	111,845.004	0.876	0.403	<0.001	0.054
LCGM6	111,695.766	0.868	0.227	<0.001	0.039
GMM					
GMM3	112,226.326	0.853	<0.001	<0.001	0.161

**Table 2:** Sample characteristics of different trajectories.

Variables	Physical Activity Trajectory (*N* = 3112)			*p* Value
	Persistently Low PA *n* = 2102/67.5%	High-Decreasing PA *n* = 508/16.3%	Moderate-Increasing PA *n* = 502/16.1%	
Age	58.97 ± 8.95	56.8 ± 7.8	54.95 ± 6.62	<0.001
Gender				<0.001
Male	855 (40.7%)	283 (55.7%)	271 (54.0%)	
Female	1247 (59.3%)	225 (44.3%)	231 (46.0%)	
Educational level				<0.001
Illiteracy	888 (42.2%)	254 (50.0%)	237 (47.2%)	
Elementary school	443 (21.1%)	116 (22.8%)	119 (23.7%)	
High school	660 (31.4%)	136 (26.8%)	141 (28.1%)	
University and above	111 (5.3%)	2 (0.4%)	5 (1.0%)	
Marital status				<0.001
With a partner	1837 (87.4%)	468 (92.1%)	476 (94.8%)	
Without a partner	265 (12.6%)	40 (7.9%)	26 (5.2%)	
Self-reported health status	2.52 ± 0.98	2.48 ± 1.0	2.59 ± 0.97	0.157
Depression	8.01 ± 6.36	8.6 ± 6.14	8.68 ± 6.32	0.032
Sleep				0.800
Insufficient Sleep	586 (27.9%)	153 (30.1%)	141 (28.1%)	
Normal Sleep	1339 (63.7%)	311 (61.2%)	323 (64.3%)	
Excessive Sleep	177 (8.4%)	44 (8.7%)	38 (7.6%)	
Social participation	1.7 ± 2.08	1.16 ± 1.69	1.24 ± 1.67	<0.001
Child support	14.72 ± 19.34	13.26 ± 16.57	10.51 ± 14.76	<0.001
Subsidies				<0.001
Without Subsidy	1534 (73.0%)	431 (84.8%)	460 (91.6%)	
Receiving Subsidy	568 (27.0%)	77 (15.2%)	42 (8.4%)	
Retirement status				<0.001
Non-Retired	1787 (85.0%)	496 (97.6%)	485 (96.6%)	
Retired	315 (15.0%)	12 (2.4%)	17 (3.4%)	
life satisfaction	3.09 ± 0.69	2.98 ± 0.67	3.0 ± 0.75	<0.001

**Table 3:** Model performance in PA trajectory classification tasks.

Comparison	Model	Accuracy	AUC	Sensitivity	PPV	Brier Score
1 vs. 2	RF	0.873 (0.863–0.881)	0.943 (0.939–0.948)	0.854 (0.841–0.865)	0.888 (0.874–0.902)	0.101 (0.098–0.104)
	XGBoost	0.854 (0.844–0.864)	0.925 (0.919–0.931)	0.820 (0.809–0.835)	0.880 (0.870–0.893)	0.105 (0.100–0.110)
	SVM	0.754 (0.739–0.771)	0.826 (0.813–0.843)	0.857 (0.833–0.880)	0.712 (0.696–0.728)	0.167 (0.159–0.174)
	KNN	0.771 (0.759–0.786)	0.864 (0.853–0.877)	0.939 (0.929–0.955)	0.703 (0.692–0.717)	0.163 (0.155–0.169)
	MLP	0.776 (0.761–0.789)	0.846 (0.832–0.857)	0.845 (0.831–0.866)	0.743 (0.729–0.757)	0.156 (0.151–0.164)
1 vs. 3	RF	0.874 (0.866–0.883)	0.945 (0.941–0.950)	0.859 (0.845–0.873)	0.886 (0.874–0.896)	0.099 (0.096–0.103)
	XGBoost	0.859 (0.850–0.867)	0.931 (0.924–0.936)	0.830 (0.820–0.840)	0.882 (0.868–0.893)	0.101 (0.097–0.107)
	SVM	0.761 (0.745–0.775)	0.832 (0.819–0.846)	0.870 (0.845–0.897)	0.715 (0.702–0.734)	0.163 (0.156–0.170)
	KNN	0.784 (0.771–0.796)	0.873 (0.861–0.883)	0.943 (0.929–0.955)	0.716 (0.702–0.728)	0.156 (0.150–0.163)
	MLP	0.786 (0.771–0.804)	0.856 (0.840–0.870)	0.856 (0.836–0.879)	0.752 (0.735–0.769)	0.151 (0.143–0.159)

Note: Trajectory 1 = persistently low PA; Trajectory 2 = high-decreasing PA; Trajectory 3 = moderate-increasing PA.

## Data Availability

The data that support the findings of this study are available in the China Health and Retirement Longitudinal Study (CHARLS) at https://charls.pku.edu.cn/en/ (accessed on 22 November 2024). The code is available on request from the corresponding author.

## Supplementary Materials

The following supporting information can be downloaded at: https://www.mdpi.com/article/10.3390/bs16071210/s1, Table S1. Baseline comparison between included and excluded participants.
