# Supplementary material for: Understanding Multilevel Correlates of Long-Term Physical Activity Trajectories Among Middle-Aged and Older Adults: A Machine-Learning Analysis
Source: Behav Sci (Basel). 2026 Jul 17;16(7):1210. doi: 10.3390/bs16071210 (PMC13405954; doi:10.3390/bs16071210)
Supplement: Supplementary file 1 [file behavsci-16-01210-s001.zip › behavsci-4349135-supplementary.pdf]

Supplementary Materials

Supplementary Table S1. Baseline comparison between included and excluded participants.

Variable	Category	Included	Excluded	p value	SMD
Age	—	57.97 ± 8.57	59.28 ± 10.44	<.001	0.129
Gender	Male	1409 (45.3%)	7013 (48.5%)	.001	0.064
	Female	1703 (54.7%)	7457 (51.5%)		
Educational level	Illiteracy	1379 (44.3%)	6445 (44.6%)	.011	0.060
	Elementary school	677 (21.8%)	3199 (22.1%)		
	High school	1004 (32.3%)	4438 (30.7%)		
	University and above	52 (1.7%)	374 (2.6%)		
Marital status	With a partner	2781 (89.4%)	12575 (86.9%)	<.001	0.075
	Without a partner	331 (10.6%)	1903 (13.1%)		
Self-reported health status	—	3.48 ± 0.98	3.50 ± 1.02	.196	0.025
Depression	—	8.21 ± 6.33	8.46 ± 6.36	.054	0.039
Sleep	Insufficient sleep	880 (28.3%)	3803 (29.3%)	.476	0.024
	Normal sleep	1973 (63.4%)	8085 (62.2%)		
	Excessive sleep	259 (8.3%)	1103 (8.5%)		
Life satisfaction	—	2.94 ± 0.70	2.95 ± 0.72	.731	0.007
Child support	—	13.80 ± 18.30	12.40 ± 18.74	<.001	0.075
Social participation	—	1.54 ± 1.97	1.34 ± 1.96	<.001	0.102
Retirement status	Non-retired	2768 (88.9%)	12307 (87.6%)	.038	0.042
	Retired	344 (11.1%)	1745 (12.4%)		
Subsidies	Without subsidy	2425 (77.9%)	10806 (75.5%)	.005	0.057
	Receiving subsidy	687 (22.1%)	3506 (24.5%)		

Note. PA variables were not compared because complete three-wave PA data were part of the inclusion criteria. The available sample size for the excluded group varied across variables because baseline information was not available for all excluded participants.
